# Recasting the Creed

**Published:** 1890-03

**Authors:** 


### HALL'S JOURNAL OF HEALTH

TRUTH DEMANDS NO SACRIFICE ; ERROR CAN MAKE NONE.

VOL. 37. MARCH, 1890. NO. 3.

### RECASTING THE CREED.

One of the most significant manifestations of the age is the open discussion, examination and deliberate repudiation by the duly ordained ministers of the Calvinistic school of theology, of some of the shockingly cruel and ungodly tenets, which since the days of cruel and dyspectic John Calvin, have stood directly in the way of all reasonable theological progress in that severe school.

Among the very worst of these tenets strictly and soberly enjoined upon every candidate for church membership were the predestination to an eternity of irrevocable misery or uninterrupted pleasure of every fellow creature born into this world; the damnation to endless torments of all those benighted races of men, who for any cause, however impossible to be avoided, failed to recognize and place their trust in the Universal Father, concerning whom they lived and died in utter ignorance: the like damnation for a like reason, of all infants whose flickering lights went out ere they took on understanding or were able to put a thought into words.

Without exception, we rejoice to be able to say that wherever these horrible hobgoblins of a "creed outworn," have been made the subject of grave donsideration by the various branches of the Presbyterian school, they have been voted to be striken from the catalogue of enjoined articles of faith.

Here are wholesome signs of progression in the most conservative of all denominational bodies; but what have these grave and reverend gentlemen to say of the absolute folly not to say wickedness, of inculcating these wretched doctrines and of enjoining them upon converts as articles of faith for nearly four hundred years? If false now, they were false in in the beginning, and whether false or true, utterly irreconcilable with the character of a merciful and loving Father, as every mother feels and knows. It is as impossible to love and adore such a monster of cruelty and injustice as to take to ones heart the monster who stains his revengeful hand with the blood of the nearest and dearest of all earthly companions. The soul abhors injustice and cruelty. There can be no division of sentiment as to them. The greater the instrument the greater the outrage, and in all those four hundred years, when the lips professed at the altar to love and reverence the Calvinistic God, the heart rebelled and gave the lips the lie and no woman fit to be the mother of her lost, child could prevent it.

There has been withal, a comical side to these discussions. Some of the participants have confessed that they never believed in the preordination and the damnation of infants and heathens. One quite young clergyman said he never felt that he could give the bereaved mother at the grave of her deceased infant any hope of its salvation? Poor soul how desperately he must love the author of such a system.

"The monstrous blasphemy of creeds
Which represent an angry God,
Who tempts man sorely through his needs,
And meets his failings with a rod—
Eternal wrath, through blood appeased,
The course of God, salvation's plan,
Are nightmare visions, which have seized
The slumbering consciousness of man."

